# IgG4-related disease presenting as a submucosal tumor of the stomach resected with laparoscopic endoscopic cooperative surgery: a case report

**DOI:** 10.1186/s40792-020-00851-8

**Published:** 2020-05-07

**Authors:** Taishi Yamane, Kojiro Eto, Takeshi Morinaga, Kazuki Matsumura, Kohei Yamashita, Ryuma Tokunaga, Kazuto Harada, Yukiharu Hiyoshi, Yohei Nagai, Masaki Iwatsuki, Shiro Iwagami, Yuji Miyamoto, Naoya Yoshida, Hideo Baba

**Affiliations:** Department of Gastroenterological Surgery, Graduate School of Life Sciences, Kumamoto University, 1-1-1 Honjo, Kumamoto, 860-8556 Japan

**Keywords:** Submucosal tumor of the stomach, IgG4-related disease (IgG4-RD), Laparoscopic endoscopic cooperative surgery (LECS)

## Abstract

**Background:**

IgG4-related disease (IgG4-RD) is an immune-mediated disorder in which abundant IgG4-positive plasma cells infiltrate affected organs. There have been reported four cases of probable IgG4-RD presenting as a submucosal tumor of the stomach. We herein report the first case of definite IgG4-RD presenting as a submucosal tumor of the stomach resected with laparoscopic endoscopic cooperative surgery (LECS).

**Case presentation:**

A 70-year-old woman with a 6-year history of autoimmune pancreatitis was referred to our department because a 15-mm submucosal tumor in the greater curvature of the lower part of the stomach had been identified via upper gastrointestinal endoscopy. Endoscopic ultrasonography showed a 10-mm low-echoic lesion derived from the submucosal layer of the stomach. A fine-needle aspiration biopsy was attempted, but the tumor was too hard for sampling. F-fluorodeoxyglucose (FDG) positron emission tomography showed an FDG uptake, suggesting a possibility of malignant disease. As the diagnosis could not be confirmed, LECS for both the diagnosis and curative treatment was performed. A histopathological examination showed a tumor with IgG4-positive lymphoplasmacytic infiltration and fibrosis. The ratio of IgG4+/IgG+ lymphoplasmacytic cells was > 80%. A laboratory examination showed elevation of the serum IgG4 levels preoperatively. Thus, the final diagnosis was IgG4-RD of the stomach. No recurrence was observed within 1 year after surgery.

**Conclusions:**

We encountered a case of definite IgG4-RD presenting as a gastric SMT in which a correct diagnosis was achieved by a minimally invasive LECS technique. IgG4-RD may present as a gastric lesion and should be taken into consideration as a differential diagnosis.

## Background

IgG4-related disease (IgG4-RD) is an immune-mediated disorder in which abundant IgG4-positive plasma cells infiltrate affected organs. This disease process is known to present in many organs and has been reported in the pancreas, biliary tree, salivary glands, kidneys, lungs, pituitary, prostate, and stomach, as well as the soft tissues, retroperitoneum, and lymph nodes [1]. There have been reported four cases of probable IgG4-RD presenting as a submucosal tumor of the stomach.

We herein report the first case of IgG4-RD that met all criteria, presenting as a submucosal tumor of the stomach, and was resected with laparoscopic endoscopic cooperative surgery (LECS).

## Case presentation

**Fig. 1:**
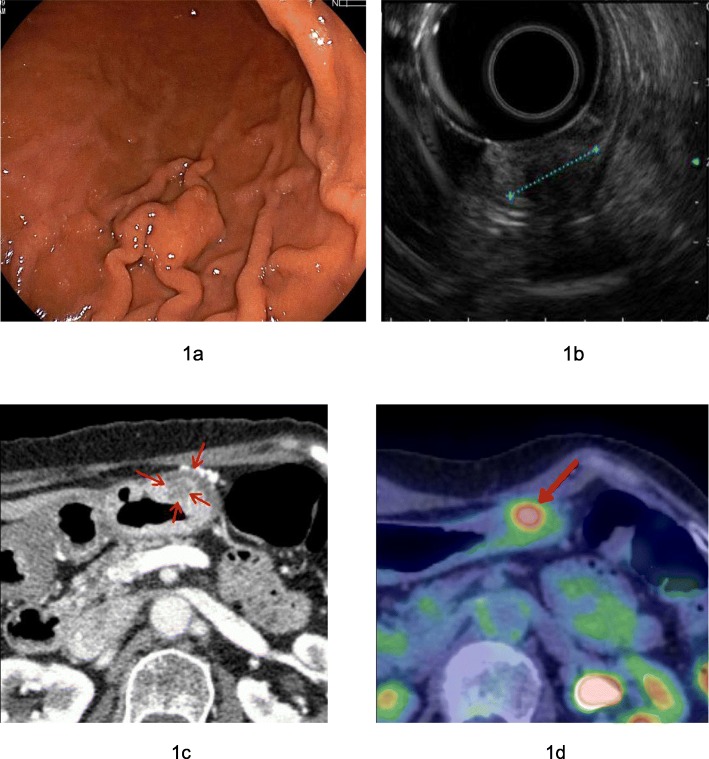
**a** Upper gastrointestinal endoscopy shows an elevated lesion in the greater curvature of the lower part of the stomach with no abnormalities of the gastric mucosa. **b** Endoscopic ultrasonography shows that the tumor is a low-echoic 10-mm lesion derived from the submucosal layer of the stomach. **c** Enhanced computed tomography shows a 15-mm hypovascular tumor in the greater curvature of the lower part of the stomach (arrows). **d** F-fluorodeoxyglucose (FDG) positron emission tomography shows an FDG uptake with an SUV_max_ of 3.8–5.1 (arrow)

A 70-year-old woman with a 6-year history of autoimmune pancreatitis was referred to our department because a gastric submucosal tumor (SMT) had been detected during screening upper gastrointestinal endoscopy. The SMT was located at the greater curvature of the lower part of the stomach and had not been noted 2 years earlier (Fig. 1a). The maximum diameter of the SMT was 15 mm. Endoscopic ultrasonography showed a 10-mm low-echoic lesion derived from the submucosal layer of the stomach (Fig. 1b).

A fine-needle aspiration biopsy was attempted, but the tumor was too hard for sampling. Enhanced computed tomography showed a 15-mm hypovascular tumor in the greater curvature of the lower part of the stomach (Fig. 1c). F-fluorodeoxyglucose (FDG) positron emission tomography showed an FDG uptake with a maximum standardized uptake value (SUV_max_) of early 3.8 to late 5.1 (Fig. 1d). A laboratory examination showed elevation of the serum IgG4 level (262 mg/dl), and all other data were within the normal range.

**Fig. 2:**
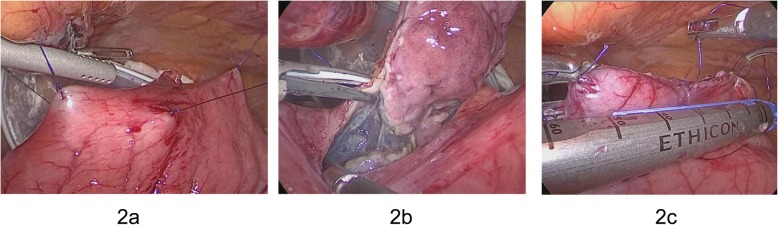
The ESD technique was performed around the tumor, and the seromuscular layer was intentionally perforated (**a**). An ultrasonically activated device was inserted into the perforation, and seromuscular dissection around the tumor was performed (**b**). The incision line was closed using a laparoscopic stapling device (**c**)

Because FDG-PET showed an FDG uptake by the tumor, we consider malignant tumor like GIST as a differential diagnosis. As the diagnosis could not be confirmed and the tumor had the possibility of malignancy, LECS was performed for both the diagnosis and curative treatment. First, endoscopic submucosal resection was performed around the tumor. The seromuscular layer was then intentionally perforated endoscopically. Finally, whole-layer resection was performed endoscopically with laparoscopic assistance, and thereafter, the remaining part of the gastric wall was ultrasonically laparoscopically resected by an activated device. After the tumor had been resected, the incision line was closed using a laparoscopic stapling device (Fig. 2a–c). The operative time and blood loss were 141 min and 5 g, respectively. The postoperative course was good, and the patient was discharged on postoperative day 7.

**Fig. 3:**
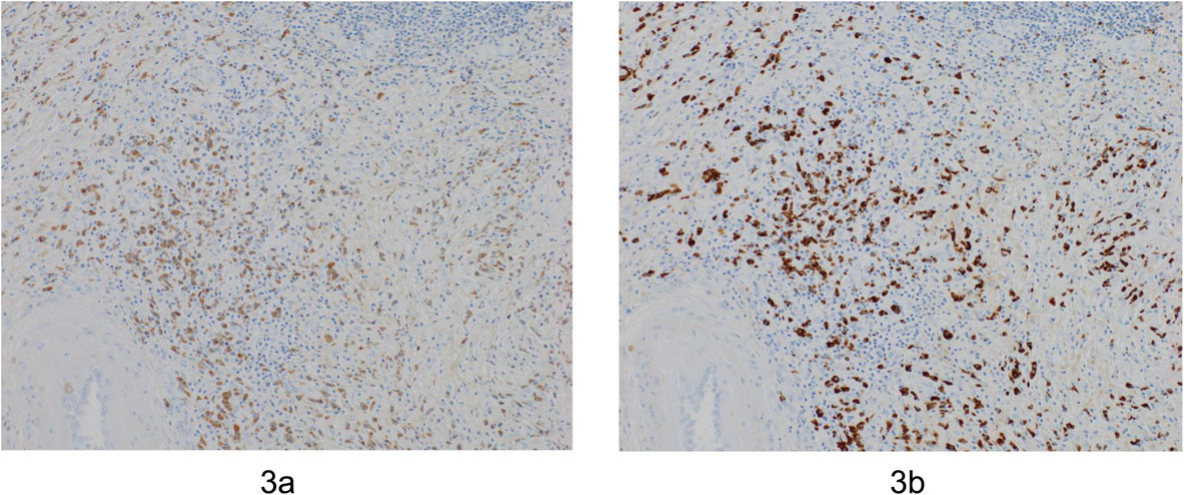
Histopathological findings show lymphoplasmacytic infiltration and fibrosis (**a**). Immunohistochemical findings show infiltration of IgG4-positive lymphoplasmacytic cells (**b**)

A histopathological examination showed lymphoplasmacytic infiltration and fibrosis (dense collagen fiber and fibroblast hyperplasia), and immunohistochemistry showed the infiltration of IgG4-positive lymphoplasmacytic cells. The ratio of IgG4+/IgG+ lymphoplasmacytic cells was > 80% (Fig. 3a, b). The final diagnosis was definite IgG4-RD of the stomach. No recurrence was observed within 1 year after surgery.

## Discussion

IgG4-RD is an immune-mediated fibro-inflammatory disease characterized by mass formation in the affected organs, elevation of serum IgG4 levels, and abundant IgG4-positive plasma cell infiltration in the affected organs. IgG4-RD covers a wide variety of diseases, including Mikulicz’s disease, autoimmune pancreatitis, Riedel thyroiditis, and retroperitoneal fibrosis [2]. The diagnostic criteria formulated in 2011 include (1) a clinical examination showing characteristic diffuse/localized swelling or masses in single or multiple organs, (2) a hematological examination showing elevated serum IgG4 concentrations (> 135 mg/dl), and (3) a histopathological examination showing marked lymphocyte and plasmacyte infiltration with fibrosis as well as IgG4+ plasma cell infiltration, with an IgG4+/IgG+ cell ratio of > 40% and > 10 IgG4+ plasma cells/high-power field [3]. If a case meets all three criteria, it is considered to be IgG4-RD, while cases that meet only 1 and 3 are considered probable IgG4-RD, and those that meet only 1 and 2 are possible IgG4-RD. A previous report described four cases of probable IgG4-RD presenting as a gastric SMT [4] (Table 1). In the present case, all of the criteria were met, so the patient was ultimately diagnosed with definite IgG4-RD.

**Table 1:** Cases of IgG4-RD presenting as a gastric SMT

Case number	Age, sex	Gastric location	Lesion type	Preoperative diagnosis	Lesion size
1	48, female	Body	Oval, smooth SMT	Round-shaped submucosal nodule	36 × 22 mm
2	56, male	Body	Round-shaped SMT	No data	8 mm
3	44, male	Body	SMT with apical ulceration	GIST	20 × 18 mm
4	29, female	Body	SMT with apical ulceration	GIST	20 × 15 mm
5 (present case)	70, female	Body	Smooth SMT	GIST	15 mm

The abovementioned four previous cases of IgG4-RD presenting as gastric SMTs were resected via wedge resection or endoscopic submucosal dissection [4], and the present case was the first case of gastric IgG4-RD resected with LECS. LECS is a procedure combining laparoscopic gastric resection and ESD for local resection of gastric tumors, and it can be performed with minimal surgical resection margins compared to conventional surgery. In this technique, incision lines are confirmed endoscopically and accurately determined by the application of an endoscopic mucosal/submucosal incision technique, while the seromuscular layer is incised laparoscopically, and the incision line is closed using a laparoscopic stapling device, resulting in minimal dissection of the normal gastric wall and minimal gastric transformation [5]. LECS is a minimally invasive technique that has proven useful for diagnostic treatment, as shown in the present tumor, in which preoperatively distinguishing between malignancy and inflammatory disease was difficult.

Since IgG4-RD have been shown to mimic malignancy both clinically and radiographically, it is important to make an accurate diagnosis prior to surgical treatment [1]. If a correct diagnosis can be obtained, IgG4-RD may respond to conservative treatment, including the use of steroids, resulting in a decreased inflammatory pseudotumor effect. However, if the preoperative diagnosis is difficult, as in the present case, minimally invasive LECS is useful for diagnostic treatment.

## Conclusions

We encountered a case of definite IgG4-RD presenting as a gastric SMT in which a correct diagnosis was achieved by a minimally invasive LECS technique. IgG4-RD may present as a gastric lesion mimicking malignancy and should be considered as a differential diagnosis.

## Data Availability

Data sharing not applicable to this article as no datasets were generated or analyzed during the current study.

## Abbreviations

IgG4-RD: IgG4-related disease
LECS: Laparoscopic endoscopic cooperative surgery
FDG: F-fluorodeoxyglucose
SMT: Submucosal tumor
SUV_max_: Maximum standardized uptake value
